# Evaluation of dronabinol to decrease opioid use for cancer-induced bone pain

**DOI:** 10.1093/oncolo/oyag163

**Published:** 2026-07-16

**Authors:** Jennifer Segar, Kiah Farr, Mary Junak, Denise Roe, Sima Ehsani, Mohab Ibrahim, Todd W Vanderah, Pavani Chalasani

**Affiliations:** Next Oncology, Houston, TX, United States; Department of Psychiatry, University of Arizona, Tucson, AZ, United States; Department of Surgery, University of Wisconsin, Madison, WI, United States; Mel and Enid Zuckerman College of Public Health, University of Arizona, Tucson, AZ, United States; Department of Biostatistics, University of Arizona Cancer Center, Tucson, AZ, United States; Department of Medicine, College of Medicine, University of Arizona, Tucson, AZ, United States; Department of Anesthesiology, College of Medicine, University of Arizona, Tucson, AZ, United States; Department of Pharmacology, College of Medicine, University of Arizona, Tucson, AZ, United States; Comprehensive Pain and Addiction Center, Tucson, AZ, United States; Department of Pharmacology, College of Medicine, University of Arizona, Tucson, AZ, United States; Comprehensive Pain and Addiction Center, Tucson, AZ, United States; Division of Hematology Oncology, The George Washington University, Washington, DC, United States; Department of Medicine, George Washington Cancer Center, Washington, DC, United States

**Keywords:** bone metastases, metastatic breast cancer, cancer-induced bone pain, opiates, biomarkers, dronabinol, CB2 agonists

## Abstract

**Background:**

Bone metastases (BM) from breast cancer cause significant cancer-induced bone pain (CIBP). Management of CIBP is primarily with opioids, which have notable side effects. In preclinical models, cannabinoid receptor (CB)2 and CB1 agonists were shown to decrease CIBP and bone degradation. We hypothesized that the addition of CB2/CB1 agonists would decrease opioid requirements in patients with BM.

**Methods:**

We conducted a single-arm study among breast cancer patients with BM on opioid therapy. Patients were treated with 10 mg dronabinol BID for 8 weeks. Our primary objective was to determine the proportion who decreased their opioid use by ≥ 20%. Participants completed the Brief Pain Inventory and the European Organization for Research and Treatment of Cancer quality of life questionnaires before and after treatment. Pre- and post-treatment blood and urine were collected for analysis of biomarkers of bone remodeling.

**Results:**

We enrolled 14 evaluable patients, and 4 decreased opioid use by ≥ 20%, meeting the primary endpoint. Patients reported significant improvements in pain severity, interference scores, quality of life, and insomnia. There was one grade 3 adverse event (dizziness) related to the study drug. A significant decrease was noted in serum C-terminal telopeptide levels with therapy.

**Conclusion:**

Our pilot study shows that the addition of dronabinol resulted in decreased opioid requirements for CIBP. Patient-reported outcomes also demonstrated improved pain and QOL with addition of dronabinol. Our results are promising and warrant further investigation into novel analgesics for CIBP to decrease opioid use.

ClinicalTrials.gov NCT03661892

Lessons learned

- Current management for CIBP involves opioids, which have many side effects.
- The addition of dronabinol resulted in decreased opioid requirements for CIBP in patients with metastatic breast cancer.
- Our results are promising and warrant further investigation of CB2 agonists for improved pain control from CIBP and reduction of opioid use.

## Drug information

**Table:** 

**DRUG INFORMATION**	
**Generic/Working name**	Dronabinol
**Drug Type**	Supportive care medicine
**Drug Class**	CB1/CB2 agonist
**Dose**	10mg BID
**Route**	PO
**Schedule of Administration: The starting dose of dronabinol was 5 mg orally twice daily for 3 days. If participants tolerated this regimen without adverse effects, the dose was increased to 10 mg in the morning and 5 mg in the evening for an additional 3 days. If further tolerated, the dose was increased to 10 mg twice daily for the rest of the study period, for a total of 8 weeks**	

**Table:** 

**TRIAL INFORMATION**	
**Disease**	Metastatic breast cancer
**Stage of disease/treatment**	IV
**Prior therapy**	On opioids for cancer related bone pain
**Type of study**	Pilot
**Primary endpoints**	To evaluate change in opiate pain medication dose after addition of dronabinol by end of 8 weeks
**Secondary endpoints**	To evaluate change in pain using the Brief Pain Inventory tool To evaluate change in quality of life using the EORTC QLQ-C30 version 3.0 questionnaire To evaluate change in biomarkers of bone modulation caused by addition of dronabinol by measuring pre- and post- dronabinol urine and serum C-terminal telopeptide (CTX) and amino terminal crosslinked telopeptides of type 1 collagen (NTX)

## Primary assessment method

**Figure 1.**
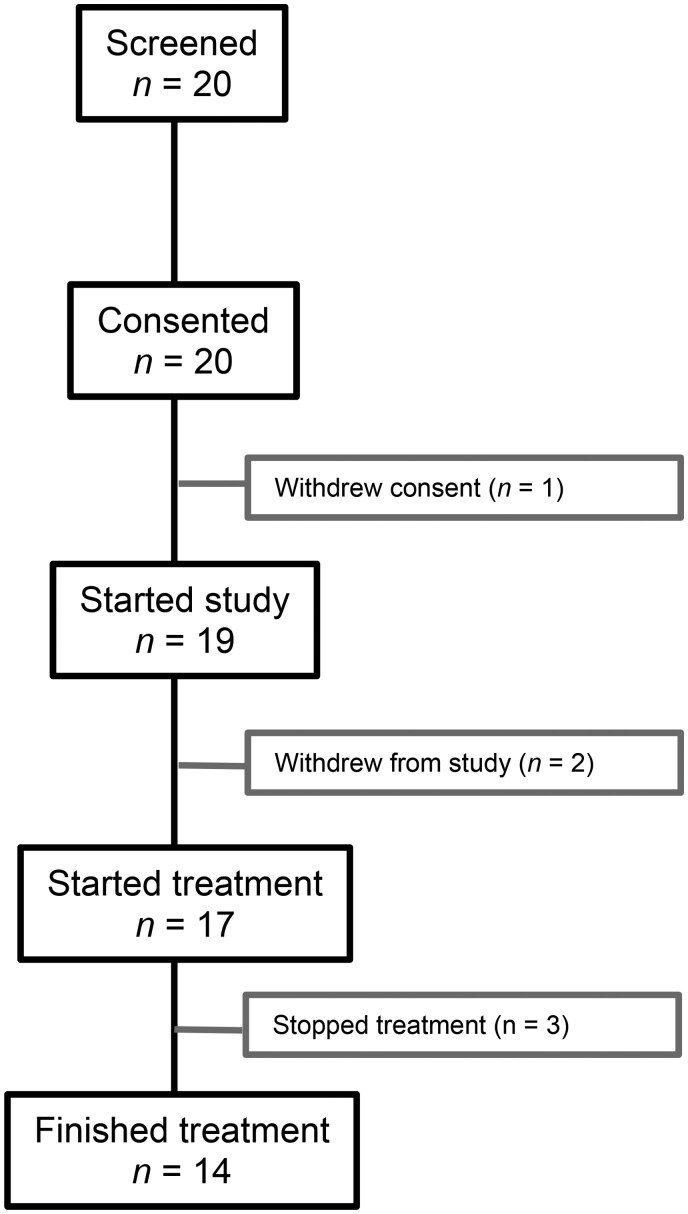
Consort diagram.

From December 2018 to July 2020, a total of 20 patients were consented, and 14 completed the study (Figure 1). Three subjects discontinued treatment due to adverse events. We included patients who stopped study treatment due to side effects in our adverse event (AE) analysis. Fourteen patients were evaluable for the primary objective (Table 1).

### Opioid use

Among the 14 evaluable patients, 6 were receiving long-acting pain medications (2 on Oxycontin, 3 on long-acting morphine and 1 using Fentanyl patch). All patients were also receiving breakthrough pain medications, with oxycodone being the most commonly prescribed (6 out of 14, 42.9%). Table 2 shows the opioid pain medication use (in MME) in week 1 and week 8. Four patients achieved a ≥ 20% reduction in opioid use by week 8.

**Table 1.** Patient characteristics.

**Demographics**	
**Total patients**	14
**Age**	
** Median**	61.5 years
** Range**	35-84 years
**Ethnicity**	
** White**	11 (78.6%)
** Hispanic**	3 (21.4%)
**Cancer therapies**	
** Chemotherapy**	3 (21.4%)
** Hormonal therapy**	4 (28.6%)
** Targeted therapies**	7 (50.0%)
**Bone-targeted agents**	
** Denosumab**	13 (92.9%)
** Zoledronic acid**	1 (7.1%)
**Tumor Biology**	
** HR+/HER2 negative**	12 (85.7%)
** HER2 positive**	1 (7.1%)
** Triple negative**	1 (7.1%)

Abbreviations: HR, hormone receptor; HER2, human epidermal growth factor Receptor 2.

**Table 2.** Total opioid dose (in MME) used by each subject in week 1 and week 8.

Subject ID	Week 1	Week 8
**1**	75 mg	0
**2**	960	1020
**3**	1830	1470
**4**	287	277
**5**	405	390
**6**	300	300
**7**	525	525
**8**	105	97.5
**9**	380	562
**10**	510	60
**11**	260	250
**12**	97.5	105
**13**	90	90
**14**	127.5	100

### Adverse events

For AE analysis, 17 subjects were included. Table 3 summarizes all the AEs. There were no grade 4 AEs, and only 1 subject had 2 grade 3 dizziness events. The most common study drug-related AEs were dizziness (*n* = 6) and cognitive disturbances (*n* = 5). Three subjects required dose reductions due to AEs. There were no study drug interruptions.

**Table 3.** Adverse events related to study drug.

Toxicity	Grade 1	Grade 2	Grade 3
**Dermatitis**	1	0	0
**Cognitive disturbance**	2	3	0
**Fatigue**	1	0	0
**Dizziness**	3	2	2
**Increased thirst**	1	0	0
**Dry mouth**	3	0	0
**Nausea**	0	2	0
**Vomiting**	1	1	0
**Diarrhea**	0	1	0
**Somnolence**	0	2	0
**Burning sensation in mouth**	1	0	0
**Anorexia**	2	0	0
**Psychosis**	1	0	0
**Myalgia**	2	1	0
**Irritability**	1	0	0
**Alopecia**	1	0	0
**Constipation**	1	0	0
**GERD**	0	1	0
**Mood alteration—Euphoria**	0	1	0
**Dyspnea**	0	1	0
**Neuropathy**	1	0	0
**Headache**	1	0	0
**Hypersomnolence**	0	1	0

### Questionnaires

#### Brief pain inventory (BPI)

There was a statistically significant improvement in pain severity and pain interference scores post-treatment. The majority of patients reported improvement in mood, sleep, and enjoyment of life with dronabinol. Raw scores are listed in Table 4.

**Table 4.** Brief pain index individual category results.

Pain metric	Improvement^a^	%	No change^a^	%	Worsened^a^	%
**Pain rating**						
** Worst pain**	4	28.6	9	64.3	1	7.1
** Least pain**	6	42.9	8	57.1	0	0
** Average pain**	5	35.7	9	64.3	0	0
** Current pain**	11	78.6	2	14.3	1	7.1
**Pain relief rating**						
** Relief from current pain management regimen**	6	42.9	8	57.1	0	0
**Pain interference rating**						
** General activity**	6	42.9	7	50	1	7.1
** Mood**	8	57.1	4	28.6	2	14.3
** Walking ability**	6	42.9	7	50	1	7.1
** Normal work**	5	35.7	9	64.3	0	0
** Relations with other people**	6	42.9	8	57.1	0	0
** Sleep**	10	71.4	3	21.4	1	7.1
** Enjoyment of life**	9	64.3	5	35.7	0	0

a Improvement defined as 2 points (or greater) difference in rating, worsened defined as 2 points (or greater) difference, no change defined as 0-1 point difference in pre and post surveys.

EORTC QLQ-C30 responses collected pre- and post-treatment demonstrated significant improvements in QOL, pain, and insomnia (Table 5 details select scores).

**Table 5.** EORTC QLQ-C30 results.^a^

Subject ID	QoL			Pain			Insomnia		
	Pre	Post	*P*-value	Pre	Post	*P*-value	Pre	Post	*P*-value
**1**	25	0	.0172	33.3	33.3	.0009	33.3	0	.1929
**2**	41.7	66.7		0	0		50	83.3	
**3**	33.3	66.7		50	0		16.7	33.3	
**4**	33.3	50		16.7	16.7		33.3	83.3	
**5**	25	66.7		16.7	16.7		0	33.3	
**6**	8.3	25		100	83.3		0	0	
**7**	58.3	50		33.3	16.7		66.7	66.7	
**8**	66.7	66.7		33.3	50		16.7	50	
**9**	58.3	66.7		0	0		66.7	1	
**10**	25	66.7		16.7	33.3		16.7	50	
**11**	50	50		16.7	50		66.7	66.7	
**12**	75	66.7		0	100		66.7	100	
**13**	25	50		16.7	16.7		16.7	33.3	
**14**	66.7	75		0	66.7		66.7	83.3	

a Select variables are represented here.

### Biomarker analysis

**Figure 2.**
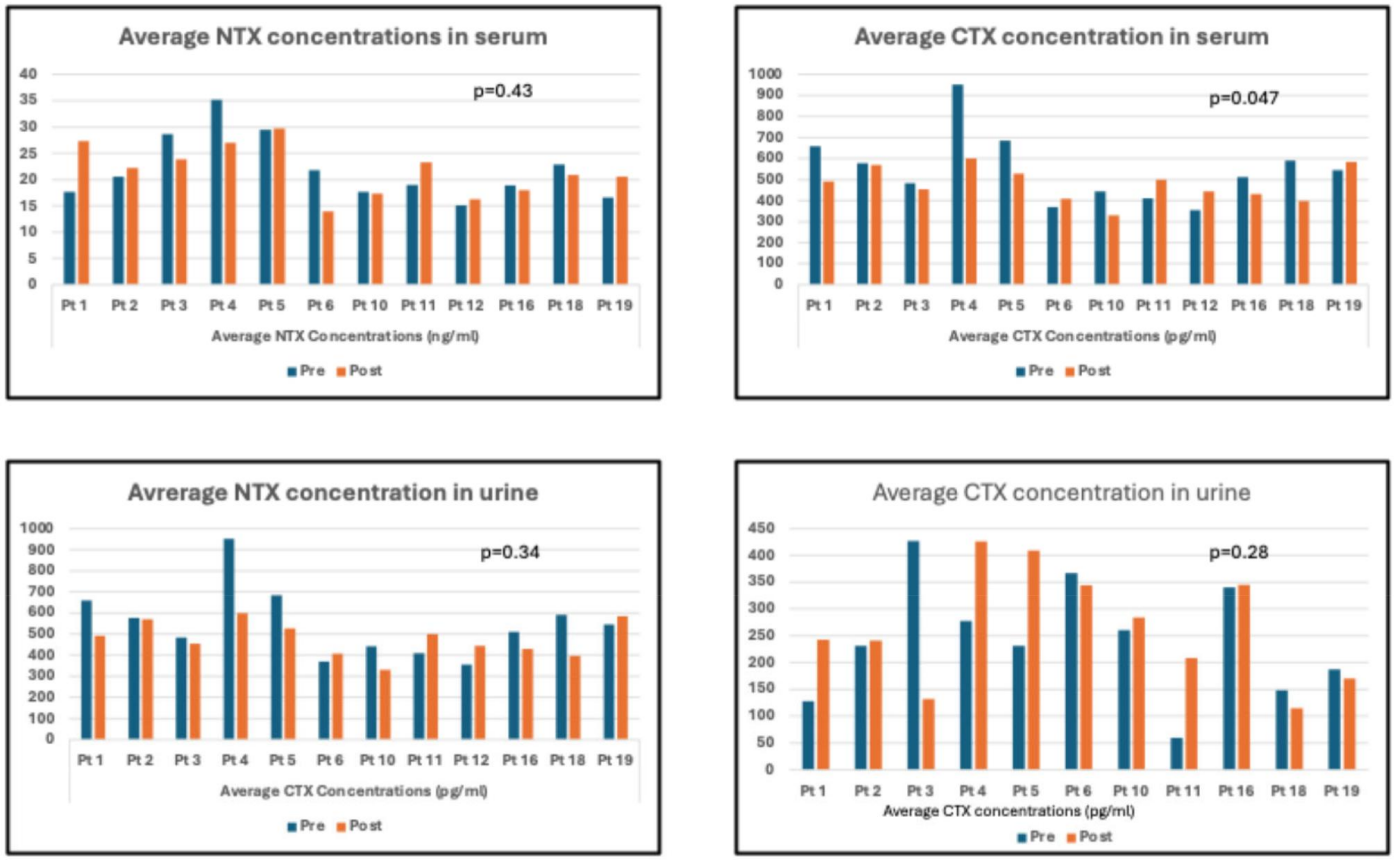
Biomarker analysis.

Figure 2 shows the pre- and post-treatment serum and urine CTX and NTX level. A significant decrease in serum CTX levels was observed following dronabinol therapy, with reductions in 7 of 12 patients. No significant differences were noted in urine CTX or serum or urine NTX levels.

### Biomarkers of bone remodeling

Both proposed biomarkers of bone remodeling, CTX and NTX, are released by osteoclast-mediated collagen degradation and increase with bone resorption. We hypothesized that serum and urine NTX and CTX would decrease with addition of dronabinol.

### Statistical analysis

The primary endpoint of this study was the proportion of women achieving at least a 20% reduction in opioid use between week 1 and week 8 of dronabinol therapy. The null hypothesis assumed that 5% of women would achieve a 20% decrease. With 14 participants, the study was powered to detect an increase from 5% to 29% (*n *= 4) with 80% power using a one-sided alpha of 0.05.

All opioid medications were assigned a milligram morphine equivalents (MME) to allow for comparison across agents. MME were calculated using Centers for Disease Control and Prevention conversion guidelines. Secondary endpoints included change in pain (assessed by BPI) and change in QOL (assessed by EORTC QLQ-C30 version 3.0). Secondary endpoints were summarized using descriptive statistics. Pre- and post-treatment questionnaire scores were compared using paired *t*-tests. Similarly, paired *t*-tests were used to evaluate differences in exploratory biomarker levels pre- and post-treatment.

## Discussion

Metastatic breast cancer commonly involves the bones, causing CIBP which significantly impacts QOL.^1^ Approved therapies for treating CIBP are limited by their side effects and may decrease QOL without improving survival or slowing disease progression.^2–13^ Opioids are the mainstay of treating CIBP; however, there are concerning preclinical data regarding long term use. It has previously been demonstrated that sustained morphine use can intensify tumor-induced pain over time and accelerate tumor-induced bone destruction.^14–16^ In mouse models, there is evidence that opioids act at the toll-like receptor 4 to mimic biological actions of lipopolysaccharide on osteoclasts and immune cells, either directly or downstream of mu opioid receptor activation promoting osteoclastogenesis.^15–18^ These changes may be counter-productive to anti-osteolytic co-therapies and pain management. Despite these concerns, opioids remain the mainstay of pain management for CIBP.

Our primary objective was met with 4 patients demonstrating a ≥ 20% reduction in opiod use after 8 weeks of dronabinol therapy. The most common AEs attributed to the study drug were dizziness and cognitive disturbance (grade 1 or 2). Patient-reported outcomes were consistent with the reduction in opioid use, with improvements in quality of life, insomnia, pain severity, and pain interference. The 3 patients who increased their opioid using during the trial period were noted to have disease progression while on study, which may have contributed to their increased opioid requirements. Of these patients with progression, 2 also reported a corresponding increase in pain severity on their BPI questionnaire. Of the 14 patients who completed the study, 9 expressed a desire to continue dronabinol therapy. Due to insurance coverage, 2 were unable to continue, while 7 patients did continue dronabinol.

In addition, recent discoveries implicate the endogenous cannabinoid system in pain modulation, bone regulation, immunity, and restraint of cancer pathogenesis. CB2 agonists lack the psychoactive effects of CB1 activators and may be preferable for clinical use in CIBP.^19^^,^^20^ Furthermore, CB2 agonists have been shown to decrease NF-κB-induced levels of several pro-inflammatory cytokines and chemokines in the bone tumor microenvironment, leading to decreased osteoclastogenesis.^21^^,^^22^ Our biomarker analysis, demonstrating a decrease in serum CTX levels by addition of dronabinol supports this finding while such difference is not noted in urine CTX and serum and urine NTX levels. Several factors may explain these findings, including small sample size, diurnal variations of CTX and NTX levels (which was not controlled for), and concurrent use of bone-targeted therapies that may influence the markers of bone resorption. The use of bone targeting therapies such as bisphosphonates and denosumab is unlikely to influence our results due to the fact that majority of the patients have been receiving these agents for more than 1 year (11 of 14 patients; the remaining 3 have received them for over 6 months). Studies show that “bone remodeling” biomarker changes with these agents occur within the first 1-3 months of therapy initiation.^23^ This suggests that the decrease observed in the serum CTX levels in our study is likely related to effects of dronabinol on bone remodeling. Larger studies are needed to further evaluate these markers. Mouse models using combined CB2 agonists and opioids have demonstrated synergistic decrease in opioid-related side effects such as nausea, vomiting, and constipation^24^^,^^25^; however, we did not observe similar findings in our QOL analysis. This may be due to the use of combined CB1/CB2 agonists, differences in side effect profiles, small sample size, or confounding effects from disease-related symptoms and breast cancer treatments.

The primary limitations of our study include the small sample size and open-label design, which may introduce subjective bias. In addition, there is a small difference in bioavailability between liquid and capsule formulations of dronabinol, with the liquid formulation being slightly more bioavailable.^26^ However, the clinical significance of this difference is unclear as, among the 4 patients who met the primary end point, 3 received liquid formulation (dispensed in 11 patients) and 1 was treated with the capsule (dispensed in 3 patients). Although there was significant improvement in QOL and BPI scores, opioid use decreased significantly in only 4 patients. The majority of patients expressed a desire to continue on dronabinol after trial completion. There may be a delayed or cumulative benefit of dronabinol leading to further decrease in opioid use, but due to the short duration of our study we were not able to capture that data. A placebo-controlled study evaluating the same formulation of dronabinol over a longer duration (≥ 6 months) may help address confounding factors such as subjective bias, disease progression, treatment changes, chemotherapy-related side effects, drug interactions and bioavailability differences, while also improving statistical power for biomarker analysis. We are currently developing such a study, as our pilot data is promising and warrants further investigation.

## Data Availability

Data are not available.
